# Supplementary material for: Quantum control using quantum memory
Source: Sci Rep. 2020 Dec 7;10:21354. doi: 10.1038/s41598-020-78455-3 (PMC7721887; doi:10.1038/s41598-020-78455-3)
Supplement: Supplementary file 1 — Supplementary Information. [file 41598_2020_78455_MOESM1_ESM.pdf]

Appendix to a "Quantum control using quantum memory" Recurrence relations

Mathieu Roget,[1,*] Basile Herzog,[2,3,†] and Giuseppe Di Molfetta[‡4,§]

[1]Aix-Marseille Université, Université de Toulon, CNRS, LIS, Marseille, France and ENS de Lyon, Département d'Informatique

[2]Aix-Marseille Université, Université de Toulon, CNRS, LIS, Marseille, France

[3]Université de Lorraine, LPCT, Nancy, France

[4]Aix-Marseille Université, Université de Toulon, CNRS, LIS, Marseille, France and Quantum Computing Center, Keio University

(Dated: November 13, 2020)

Let's recall the initial state :

$$\Psi(0) = |0\rangle\,|v^-\rangle \left(\bigotimes_{x=-\lfloor \frac{N}{2} \rfloor}^{-1} A_{-x}\,|0\rangle + B_{-x}\,|1\rangle \right) \left(\bigotimes_{x=0}^{\lfloor \frac{N}{2} \rfloor} |0\rangle \right),$$

and let's rephrase it in the following array :

position	..	-2	-1	0	1	2	..
velocity	$\begin{pmatrix} .. \\ .. \end{pmatrix}$	$\begin{pmatrix} 0 \\ 0 \end{pmatrix}$	$\begin{pmatrix} 0 \\ 0 \end{pmatrix}$	$\begin{pmatrix} 1 \\ 0 \end{pmatrix}$	$\begin{pmatrix} 0 \\ 0 \end{pmatrix}$	$\begin{pmatrix} 0 \\ 0 \end{pmatrix}$	$\begin{pmatrix} .. \\ .. \end{pmatrix}$
memory	$\begin{pmatrix} .. \\ .. \end{pmatrix}$	$\begin{pmatrix} A_2 \\ B_2 \end{pmatrix}$	$\begin{pmatrix} A_1 \\ B_1 \end{pmatrix}$	$\begin{pmatrix} 1 \\ 0 \end{pmatrix}$	$\begin{pmatrix} 1 \\ 0 \end{pmatrix}$	$\begin{pmatrix} 1 \\ 0 \end{pmatrix}$	$\begin{pmatrix} .. \\ .. \end{pmatrix}$

a. First time step :
Q can only act on $x = 0$, on which there is $|v^-\rangle$ associated to its left and right neighbors which are in memory space $A_1\,|0\rangle + B_1\,|1\rangle$ (left neighbor in position $x = -1$) and $1 \times |0\rangle$ (right neighbor in position $x = +1$).
So that Q will act on $A_1\,|v^-00\rangle + B_1\,|v^-10\rangle$ and

$$Q\left(A_1\,|v^-00\rangle + B_1\,|v^-10\rangle\right) = A_1\,|v^-00\rangle + B_1\,|v^+01\rangle\,.$$

Now, the shift acts in the position velocity space on $A_1\,|0\rangle\,|v^-\rangle + B_1\,|0\rangle\,|v^+\rangle$, and its action is

$$S\left(A_1\,|0\rangle\,|v^-\rangle + B_1\,|0\rangle\,|v^+\rangle\right) = A_1\,|{-1}\rangle\,|v^-\rangle + B_1\,|{+1}\rangle\,|v^+\rangle\,.$$

Finally, the state after first iteration :

position	..	-2	-1	0	1	2	..
velocity	$\begin{pmatrix} .. \\ .. \end{pmatrix}$	$\begin{pmatrix} 0 \\ 0 \end{pmatrix}$	$\begin{pmatrix} A_1 \\ 0 \end{pmatrix}$	$\begin{pmatrix} 0 \\ 0 \end{pmatrix}$	$\begin{pmatrix} 0 \\ B_1 \end{pmatrix}$	$\begin{pmatrix} 0 \\ 0 \end{pmatrix}$	$\begin{pmatrix} .. \\ .. \end{pmatrix}$
memory	$\begin{pmatrix} .. \\ .. \end{pmatrix}$	$\begin{pmatrix} A_2 \\ B_2 \end{pmatrix}$	$\begin{pmatrix} 1 \\ 0 \end{pmatrix}$	$\begin{pmatrix} 1 \\ 0 \end{pmatrix}$	$\begin{pmatrix} 1 \\ 1 \end{pmatrix}$	$\begin{pmatrix} 1 \\ 0 \end{pmatrix}$	$\begin{pmatrix} .. \\ .. \end{pmatrix}$

In position velocity space :

$$\psi(1) = A_1\,|{-1}\rangle\,|v^-\rangle + B_1\,|{+1}\rangle\,|v^+\rangle\,,$$

So that

$$\psi^-_{-1}(1) = A_1 \times 1 = A_{-x}\psi^-_{x+1}(0)$$

and

$$\psi^+_1 = B_1 \times 1 = B_{-x+2}\psi^-_{x-1}(0).$$

From there, the recurrence relations for the amplitudes $\psi^\pm_x(t)$ reads :

$$\psi^-_x(t+1) = A_{-x}\psi^-_{x+1}(t),$$
$$\psi^+_x(t+1) = B_{-x+2}\psi^-_{x-1}(t).$$

b. Second time step :
Now Q acts on $x = -1$ and $x = 1$ where, for $A_1\,|x=-1\rangle\,|v^-\rangle$, we have for the left neighbor $A_2\,|0\rangle + B_2\,|1\rangle$ and for the right neighbor $1 \times |0\rangle$, so that Q will act there on $A_2A_1\,|v^-00\rangle + B_2A_1\,|v^-10\rangle$, resulting in

$$Q\left(A_2A_1\,|v^-00\rangle + B_2A_1\,|v^-10\rangle\right) = A_2A_1\,|v^-00\rangle + B_2A_1\,|v^+01\rangle\,.$$

For $B_1\,|x=+1\rangle\,|v^+\rangle$, we have for the left neighbor $1 \times |0\rangle$ and for the right neighbor $1 \times |0\rangle$, so that Q will act on $B_1\,|v^+00\rangle$, and

$$QB_1\,|v^+00\rangle = B_1\,|v^+11\rangle\,.$$

Now the shift will act, in the position velocity space, as

$$\begin{aligned} &S\left(A_2A_1\,|{-1}\rangle\,|v^-\rangle + B_2A_1\,|{-1}\rangle\,|v^+\rangle + B_1\,|{+1}\rangle\,|v^+\rangle\right) \\ &= A_2A_1\,|{-2}\rangle\,|v^-\rangle + B_2A_1\,|0\rangle\,|v^+\rangle + B_1\,|{+2}\rangle\,|v^+\rangle\,. \end{aligned}$$

For the amplitudes $\psi^\pm$, this gives :

$$\psi^-_{-2}(2) = A_2 \times A_1 = A_{-x}\psi^-_{x+1}(1),$$

$$\psi^+_0(2) = B_2 \times A_1 = B_{-x+2}\psi^-_{x-1}(1),$$

$$\psi^+_2(2) = B_1 = \psi^+_{x-1}(1).$$

So that now the full recurrence relations are:

$$\psi^-_x(t+1) = A_{-x}\psi^-_{x+1}(t)$$
$$\psi^+_x(t+1) = \psi^+_{x-1}(t) + B_{-x+2}\psi^-_{x-1}(t).$$
